# Supplementary material for: Do biomolecular condensates regulate the transcriptional and post-transcriptional responses of plant roots to water deficit?
Source: Quant Plant Biol. 2025 Nov 12;6:e40. doi: 10.1017/qpb.2025.10032 (PMC12722080; doi:10.1017/qpb.2025.10032)
Supplement: Masson et al. supplementary material [file S2632882825100325sup001.pdf]

Supplemental material to Masson et al., Do biomolecular condensates regulate the transcriptional and post-transcriptional responses of plant roots to water deficit?

Table S1: Range of experimental values for elastic modulus, turgor pressure and osmotic pressure, for root cortical cell of different species.

Elastic modulus (MPa)	1 to 4	Arabidopsis Thaliana (Javot *et al.*, 2003), Phaseolus coccineus (Steudle & Brinckmann, 1989), Zea mays (Ding et al., 2020), Barley (Knipfer et al., 2021), Soybean (Vandeleur et al., 2014)
Initial turgor pressure (Mpa)	0.18 to 0.60	Arabidopsis Thaliana (Javot et al., 2003; Crabos et al., 2023), Zea mays (Ding et al., 2020; Rygol et al., 1993), Aster tripolium (Zimmermann et al., 1992)

Table S2 : Osmotic stress found in the literature related to condensate or speckles.

Reference	Ψ (MPa)	Osmoticum
(Crabos et al., 2023)	-0.073 to -0.516	25 to 100 mM NaCl<br>50 to 150 mM Sorbitol<br>75 to 150 $g.l^{-1}$ PEG8000
(Z. Wang et al., 2024)	-0.37 to -1.39	150, 225, 300 mM Mannitol<br>150 and 300 mM NaCl<br>24% PEG8000
(Chong et al., 2019)	-0.7 and -1.2	PEG
(B. Wang et al., 2022)	-0.74 and -1.48	300 mM NaCl<br>300 mM Mannitol
(Dorone et al., 2021)	-0.99 to -10	200 to 2000 mM NaCl
(Khan et al., 2014)	-0.99	200 mM NaCl
(Soma et al., 2020)	-1.24	250 mM NaCl<br>500 mM Mannitol
(Cao et al., 2023)	-1.98	400 mM NaCl

Note S1

To make the schematic representations P_t and Π versus Ψ, we first calculated all variables versus the relative cell volume V/V_0 using equations 2, 3 and 4, 5 then we plotted variables versus water potential (eqn 6). For the sake of simplicity, an initial outer water potential was considered to zero, and a cell at the osmotic equilibrium, i.e. Ψ=0. Therefore, initial turgor and osmotic pressure were equals. Calculation was performed using data obtained with the cell pressure probe on Arabidopsis root cortical cells, ϵ=2.23MPa (Javot et al., 2003) and P=0.41MPa (Crabos et al., 2023 and table S1). The turgor pressure decreases from P_{t0} to 0 at the point of incipient plasmolysis $V/V_0 = exp(-P_{t0}/\epsilon)$=0.86. From this point there is no turgor pressure anymore, the water potential equals the osmotic potential $\Psi_{\Pi}=-\Pi$.

The segments and points at the bottom of the figure were extracted from studies of in vivo biomolecular condensate formation under hyper-osmotic shock (see Table S2).

Details of P_t and Π versus Ψ calculations:

Neglecting gravitational component, the water potential in the cytosol is simply given by:

$$\Psi = P_t - \Pi \tag{1}$$

with P_t the turgor pressure, σ the reflection coefficient and Π the osmotic pressure. The turgor pressure is the excess of hydrostatic pressure inside the protoplast regarding the ambient atmospheric pressure. It is built up by the plasma membrane pressing the cell wall. If for instance water leave the cell, the protoplast volume will decrease as the turgor pressure, inversely if water enter the cytosol. Before plasmolysis the turgor pressure variation is given by the following equation:

$$dP_t = \epsilon \frac{dV}{V} \tag{2}$$

with V the cell volume and ϵ the elastic modulus. Therefore, P_t may be calculated according to the relative variation of cell volume, and considering an initial state of V_0 and P_{t0}, as follow:

$$P_t = \epsilon ln\left(\frac{V}{V_0}\right) + P_{t0} \tag{3}$$

From the point of incipient plasmolysis, the turgor pressure can be approximated as zero. This point is at $V/V_0 = exp(-P_{t0}/\epsilon)$. According to Table S1 V/V_0 would range from 0.55 to 0.96.

The osmotic pressure is given by the Van't Hoff relation:

$$\Pi = RT \sum C_j \tag{4}$$

with R the gas constant, T the temperature and C_j the molality of the solutes. Considering one non-permeating solute in the cytosol, C and so Π, may be calculated from the relative volume as follow:

$$\frac{C}{C_0} = \frac{\Pi}{\Pi_0} = \frac{V_0}{V} \tag{5}$$

Finally, the water potential may be calculated according to the cell volume:

$$\begin{cases} \Psi = \epsilon ln\left(\frac{V}{V_0}\right) + P_{t0} - \Pi_0 \frac{V_0}{V} & \forall\, P_t \geq 0 \\ \Psi = -\Pi_0 \frac{V_0}{V} & \forall\, P_t < 0 \end{cases} \tag{6}$$

Bibliography

Cao, X., Du, Q., Guo, Y., Wang, Y., & Jiao, Y. (2023). Condensation of STM is critical for shoot meristem maintenance and salt tolerance in *Arabidopsis*. *Molecular Plant*, *16*(9), 1445–1459. https://doi.org/10.1016/j.molp.2023.09.005

Chong, G. L., Foo, M. H., Lin, W.-D., Wong, M. M., & Verslues, P. E. (2019). Highly ABA-Induced 1 (HAI1)-Interacting protein HIN1 and drought acclimation-enhanced splicing efficiency at intron retention sites. *Proceedings of the National Academy of Sciences*, *116*(44), 22376–22385. https://doi.org/10.1073/pnas.1906244116

Crabos, A., Huang, Y., Boursat, T., Maurel, C., Ruffel, S., Krouk, G., & Boursiac, Y. (2023a). Distinct early transcriptional regulations by turgor and osmotic potential in the roots of Arabidopsis. *Journal of Experimental Botany*, *74*(18), 5917–5930. https://doi.org/10.1093/jxb/erad307

Ding, L., Milhiet, T., Couvreur, V., Nelissen, H., Meziane, A., Parent, B., Aesaert, S., Lijsebettens, M. V., Inzé, D., Tardieu, F., Draye, X., & Chaumont, F. (2020). Modification of the Expression of the Aquaporin ZmPIP2;5 Affects Water Relations and Plant Growth. *Plant Physiology*, *182*(4), 2154–2165. https://doi.org/10.1104/pp.19.01183

Dorone, Y., Boeynaems, S., Flores, E., Jin, B., Hateley, S., Bossi, F., Lazarus, E., Pennington, J. G., Michiels, E., De Decker, M., Vints, K., Baatsen, P., Bassel, G. W., Otegui, M. S., Holehouse, A. S., Exposito-Alonso, M., Sukenik, S., Gitler, A. D., & Rhee, S. Y. (2021). A prion-like protein regulator of seed germination undergoes hydration-dependent phase separation. *Cell*, *184*(16), 4284-4298.e27. https://doi.org/10.1016/j.cell.2021.06.009

Javot, H., Lauvergeat, V., Santoni, V., Martin-Laurent, F., Güçlü, J., Vinh, J., Heyes, J., Franck, K. I., Schäffner, A. R., Bouchez, D., & Maurel, C. (2003). *Role of a Single Aquaporin Isoform in Root Water Uptake*. *15*, 509–522. https://doi.org/10.1105/tpc.008888

Khan, A., Garbelli, A., Grossi, S., Florentin, A., Batelli, G., Acuna, T., Zolla, G., Kaye, Y., Paul, L. K., Zhu, J.-K., Maga, G., Grafi, G., & Barak, S. (2014). The Arabidopsis STRESS RESPONSE SUPPRESSOR DEAD-box RNA helicases are nucleolar- and chromocenter-localized proteins that undergo stress-mediated relocalization and are involved in epigenetic gene silencing. *The Plant Journal*, *79*(1), 28–43. https://doi.org/10.1111/tpj.12533

Knipfer, T., Danjou, M., Vionne, C., & Fricke, W. (2021). Salt stress reduces root water uptake in barley (Hordeum vulgare L.) through modification of the transcellular transport path. *Plant Cell Environ*, *44*(2), 458-475-458–475. https://doi.org/10.1111/pce.13936

Rygol, J., Pritchard, J., Zhu, J. J., Tomos, A. D., & Zimmermann, U. (1993). Transpiration Induces Radial Turgor Pressure Gradients in Wheat and Maize Roots. *Plant Physiology*, *103*(2), 493–500. https://doi.org/10.1104/pp.103.2.493

Soma, F., Takahashi, F., Suzuki, T., Shinozaki, K., & Yamaguchi-Shinozaki, K. (2020). Plant Raf-like kinases regulate the mRNA population upstream of ABA-unresponsive SnRK2 kinases under drought stress. *Nature Communications*, *11*(1), 1373. https://doi.org/10.1038/s41467-020-15239-3

Steudle, E., & Brinckmann, E. (1989). The Osmometer Model of the Root: Water and Solute Relations of Roots of Phaseolus coccineus. *Botanica Acta*, *102*(1), 85–95. https://doi.org/10.1111/j.1438-8677.1989.tb00071.x

Vandeleur, R. K., Sullivan, W., Athman, A., Jordans, C., Gilliham, M., Kaiser, B. N., & Tyerman, S. D. (2014). Rapid shoot-to-root signalling regulates root hydraulic conductance via aquaporins. *Plant, Cell & Environment*, *37*(2), 520–538. https://doi.org/10.1111/pce.12175

Wang, B., Zhang, H., Huai, J., Peng, F., Wu, J., Lin, R., & Fang, X. (2022). Condensation of SEUSS promotes hyperosmotic stress tolerance in Arabidopsis. *Nature Chemical Biology*, *18*(12), Article 12. https://doi.org/10.1038/s41589-022-01196-z

Wang, Z., Yang, Q., Zhang, D., Lu, Y., Wang, Y., Pan, Y., Qiu, Y., Men, Y., Yan, W., Xiao, Z., Sun, R., Li, W., Huang, H., & Guo, H. (2024). A cytoplasmic osmosensing mechanism mediated by molecular crowding–sensitive DCP5. *Science*, *386*(6721), eadk9067. https://doi.org/10.1126/science.adk9067

Zimmermann, U., Rygol, J., Balling, A., Klock, G., Metzler, A., & Haase, A. (1992). Radial Turgor and Osmotic Pressure Profiles in Intact and Excised Roots of Aster tripolium: Pressure Probe Measurements and Nuclear Magnetic Resonance-Imaging Analysis. *PLANT PHYSIOLOGY*, *99*(1), 186–196. https://doi.org/10.1104/pp.99.1.186
